# Hardship at birth alters the impact of climate change on a long-lived predator

**DOI:** 10.1038/s41467-022-33011-7

**Published:** 2022-09-27

**Authors:** Fabrizio Sergio, Giacomo Tavecchia, Julio Blas, Alessandro Tanferna, Fernando Hiraldo, Erkki Korpimaki, Steven R. Beissinger

**Affiliations:** 1 Department of Conservation Biology, Estación Biológica de Doñana - CSIC, 41092 Seville, Spain; 2 Population Ecology Group, Institute for Mediterranean Studies (IMEDEA), CSIC-UIB, 07190 Esporles, Spain; 3 Section of Ecology, Department of Biology, University of Turku, FI-20014 Turku, Finland; 4 Department of Environmental Science, Policy & Management, University of California, Berkeley, 94720 CA USA; 5 Museum of Vertebrate Zoology, University of California, Berkeley, 94720 CA USA

**Keywords:** Climate-change ecology, Conservation biology

## Abstract

Climate change is increasing the frequency of extreme events, such as droughts or hurricanes, with substantial impacts on human and wildlife communities. Extreme events can affect individuals through two pathways: by altering the fitness of adults encountering a current extreme, and by affecting the development of individuals born during a natal extreme, a largely overlooked process. Here, we show that the impact of natal drought on an avian predator overrode the effect of current drought for decades, so that individuals born during drought were disadvantaged throughout life. Incorporation of natal effects caused a 40% decline in forecasted population size and a 21% shortening of time to extinction. These results imply that climate change may erode populations more quickly and severely than currently appreciated, suggesting the urgency to incorporate “penalties” for natal legacies in the analytical toolkit of impact forecasts. Similar double impacts may apply to other drivers of global change.

## Abstract (web summary)

The long-term effects of extreme climate events in early life are largely overlooked in forecasts of climate change impacts. Here, the authors show that raptorial red kites born during drought are disadvantaged throughout life, and including this climate legacy leads to substantial decreases in forecasted population size and time to extinction.

## Introduction

Climate change is causing an increase in the frequency and magnitude of extreme weather events^1,2^. These events can trigger or function as major ecological disturbances, such as droughts, fires or hurricanes, capable of restructuring entire ecosystems^3^. Expectedly, such increase in climate-driven disturbances has attracted exponential research-attention^4^, given its far-reaching and frequently dramatic impacts on human society and on plant and animal individuals, populations and communities^4–9^. However, relatively few studies have elucidated the demographic mechanisms that mediate responses to extreme events, a crucial step to develop predictions of future outcomes^10^.

**Fig. 1:**
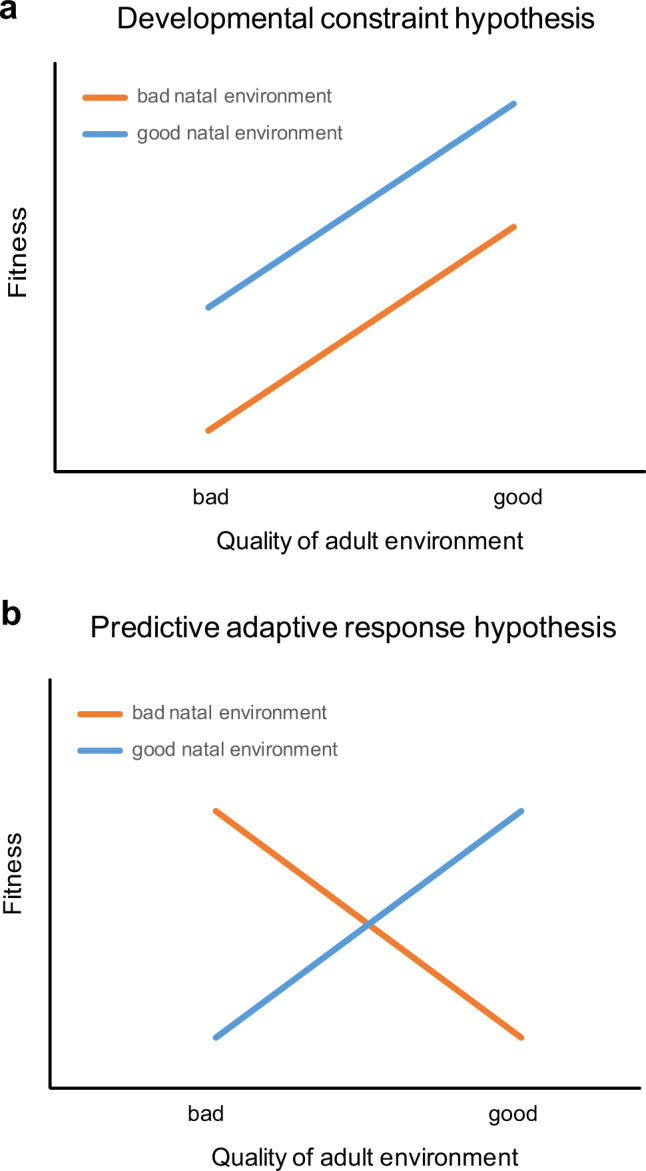
Two main hypotheses examine the fitness effects of the interplay between conditions experienced during development and those encountered later in life. In the “developmental constraint” or “silver spoon” hypothesis (**a**), harsh conditions during early development constrain phenotype quality, undermining its future fitness, so that phenotypes generated in favourable settings are always superior to those generated under hardship regardless of the environment experienced later in life. Thus, for all levels of environmental quality encountered in adult life (x-axis), the fitness of individuals that experienced favourable (natal) conditions during development is always higher than the fitness of those that experienced hardship during development (parallel lines of **a**). In the “predictive adaptive response” hypothesis (PAR, **b**) individuals enjoy a fitness advantage when they encounter as adults the same environmental quality that they experienced during development. Thus, for example, the fitness of individuals that experienced hardship during development is inferior to the one of other individuals when adult conditions are favourable, but superior when adult conditions are again challenging. This results in the crossing lines of **b**. Readapted from refs. 12,28,70.

One highly overlooked aspect in this context is the fact that each extreme event can affect a population through two simultaneous pathways: (1) by altering the vital rates and fitness of adult individuals encountering the (current) extreme event, and/or (2) by affecting the developmental stage of the cohort of juvenile individuals born during the (natal) extreme event. While many studies have focused on the former, the latter pathway has been almost completely overlooked. This is peculiar, given that conditions experienced during development can affect an individual’s demographic performance for the rest of its life, an effect that has been recognized for more than twenty years^11,12^. For example, individuals born in poor quality habitats, stressful environments or years of low food availability show impaired survival and reproduction in adult life in various fish, reptile, avian and mammal species, including humans^13–22^. In particular, two hypotheses borrowed from the field of life history evolution and human medicine are particularly relevant in the context of increasing frequencies of climate extremes, as they integrate the interplay between environmental conditions experienced during early development and during later adult life (Fig. 1). (1) The “predictive adaptive response” hypothesis (PAR) posits that unfavourable conditions experienced during early development could confer phenotypic advantages enjoyed later in adult life if the hardship continues or when the individual again encounters the unfavourable environment^23,24^. For example, a phenotype characterized by smaller body size induced by nutritional deficits during development could be selectively advantaged during a future famine through its lower nutritional needs. (2) Conversely, the “developmental constraint”, or “silver spoon” hypothesis postulates that harsh conditions during early development constrain phenotype quality, undermining future fitness, so that phenotypes generated in favourable settings are always superior to those generated under hardship, regardless of the environment experienced later in life^12,25^. The two hypotheses produce contrasting predictions: an unfavourable extreme episode experienced during development could either enhance or weaken the capability of an individual to cope with similar conditions, should they reappear or become more frequent during adulthood, as predicted by many climate change projections^1,2,9^.

Both hypotheses have two major implications. First, early conditions could set individuals on different life history trajectories, increasing heterogeneity in individual quality, with subsequent repercussions on population viability and fitness^16,26,27^. Second, early hardship could generate a set of individuals that will perform poorly under certain (or all) conditions, thus weakening the overall population. Both aspects could alter the predictions of future climate impacts if models discount the long-term effects of early conditions during development on population processes. In particular, should extreme events negatively affect adults over the short-term and developing juveniles over the long-term, this would represent a “double impact” to the population and be more debilitating than a simple direct effect on adults. Therefore, there is an urgent need for studies that integrate the direct impact of extreme events on adult individuals with their long-term impact from early developmental effects.

Here, we examine the demographic response to drought of a long-lived avian predator, the red kite *Milvus milvus* (hereafter “kite”; maximum longevity = 30 years). We show that drought conditions experienced at birth may be more important determinants of survival than the conditions encountered as an adult decades later, with profound population repercussions.

## Results & discussion

### Terminology

Hereafter, for simplicity we define (i) non-drought years as “normal years”, (ii) the conditions experienced during development and their effects as “natal conditions and effects”, and (iii) those encountered in adult life as “contemporary or current conditions and effects”. Thus, for example, natal drought is the one that a 12-year old individual experienced in its birth year (12 years before), while a current drought would be the one that it encounters during its 12th year of age. We also define the birth order in a brood as “brood rank”, with dominant, 1st born nestlings defined as Rank 1 individuals and their 2nd and 3rd born siblings as Rank 2 individuals, which frequently suffer sibling aggression and food deprivation (see Study species for details). The PAR and “silver spoon” hypotheses should be assessed by comparing effects within individuals (e.g. on individuals of the same rank).

### Drought impact on the food-base

**Fig. 2:**
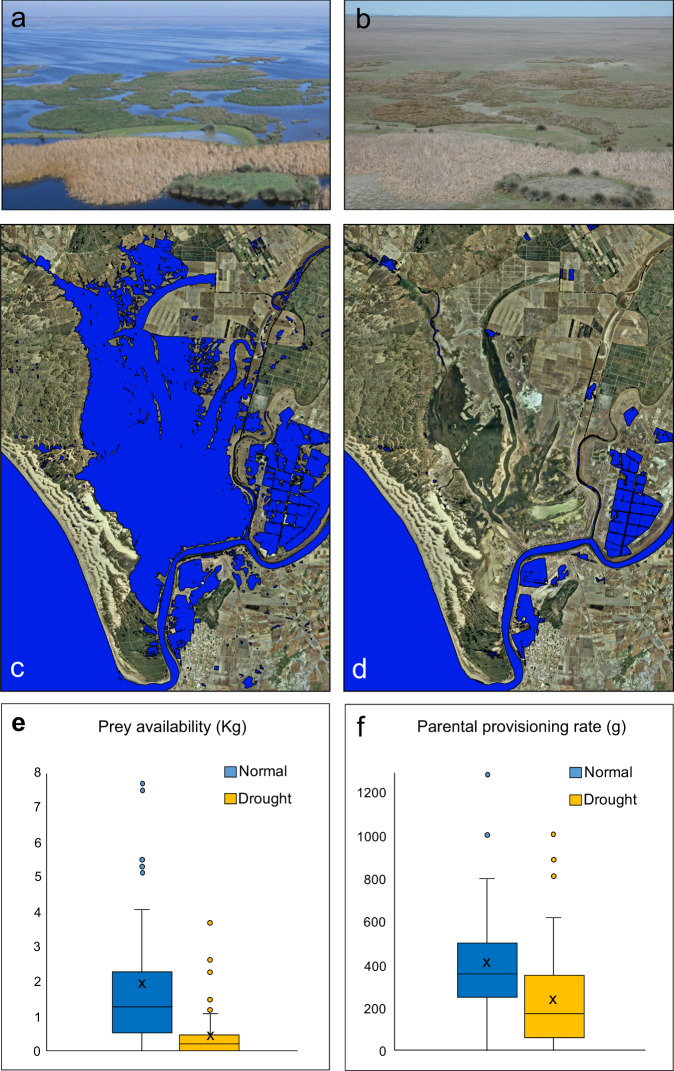
Impact of drought on the wetland ecosystem of Doñana National Park (southwest Spain) and on its prey-base available to red kites. Drought conditions (**b**, **d**) convert the large wetland of Doñana (**a**, **c**) into an arid steppe (**b**, **d**), imposing a severe reduction in prey availability (**e**), which translated into lower parental provisioning rates to the offspring (**f**). Prey availability is the cumulative mass of prey counted during 120 prey transects (*N* = 60 transects sampled during drought and 60 transects sampled during normal years; see Methods), while parental provisioning is the daily cumulative biomass of prey delivered by the parents per offspring, as assessed by 97 days of camera-trapping at the nest (*N* = 51 days sampled during drought and 46 during normal years). Symbols: in each boxplot, the centre line represents the median, the “X” symbol the mean value, the length of the box extends through the interquartile range (IQR) and the whiskers extend to 1.5 × IQR. All data points outside this range are plotted individually. Source data are provided as a Source Data file.

Drought imposed a 4.5-fold decrease in available prey biomass (Fig. 2e and Supplementary Table 1) and a 1.7-fold decrease in food provisioning rates to the nestlings by breeding parents (Fig. 2f, Supplementary Table 1). The lower impact on provisioning than available prey suggests some capability for compensation by the parents, likely through increased foraging effort per food item captured. Thus, drought likely functioned as a nutritional bottleneck for both offspring (through diminished prey deliveries per nestling) and parents (through lowered prey availability and, possibly, increased hunting effort).

### Drought impact on breeding performance

**Fig. 3:**
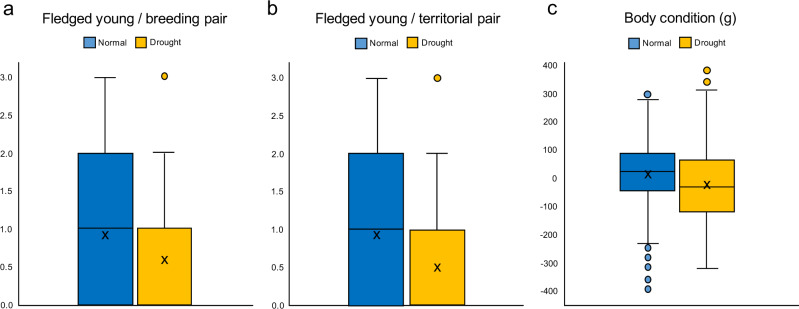
Impact of drought on the number and quality of the offspring generated by the red kite population of Doñana National Park (southwest Spain). Drought depressed the number of fledged young (**a**, **b**; *N* = 455 and 181; and *N* = 840 and 316 breeding attempts for normal and drought years, respectively), as well as their body condition (**c**; mass deficit: observed mass of a nestling compared to the mass expected for its age, see Methods; *N* = 273 and 94). Symbols: in each boxplot, the centre line represents the median, the “X” symbol the mean value, the length of the box extends through the interquartile range (IQR) and the whiskers extend to 1.5 × IQR. All data points outside this range are plotted individually. Source data are provided as a Source Data file.

Except for clutch size and hatching success, all components of breeding performance declined strongly with drought, which caused a 126 % increase in the probability of skipping reproduction, a 56 % increase in the rate of brood reduction, a 229 % increase in nest predation, and a 46 % and 37 % reduction in the number of fledglings raised per territorial and breeding pair, respectively (Fig. 3, Supplementary Fig. 1 and Supplementary Table 1). Lower provisioning rates during drought resulted in a 330 % reduction in nestling body condition (Fig. 3, Supplementary Table 1), but no detectable effect on body size (Supplementary Table 1).

### Drought impact on survival

**Fig. 4:**
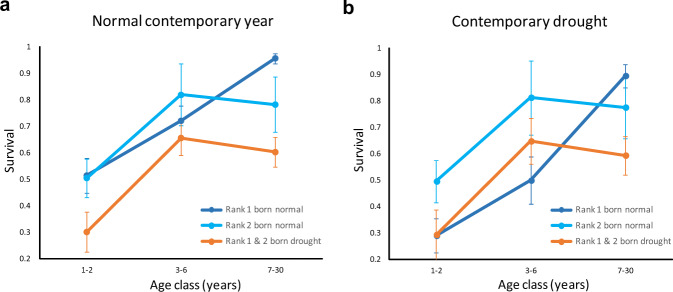
Impact of natal and current drought on the survival of the red kite population of Doñana National Park (southwest Spain). Survival through life of firstborn (Rank 1) and junior (Rank 2) red kite nestlings born in years of normal marsh inundation (dark blue and light blue lines for Rank 1 and Rank 2 individuals, respectively) or drought (orange lines), and when they encountered in later life a contemporary drought (**b**) or a contemporary year of normal marsh inundation (**a**). Main results: (1) in adults (above 7 years old, right-most portion of both panels), natal drought depressed survival for all individuals, irrespective of current conditions (compare blue vs orange lines within each panel); (2) natal drought lowered survival for most individuals at all ages (compare light blue vs orange lines), except for Rank 1 individuals born in normal years (dark blue line). (3) The latter suffered increased mortality when they encountered drought during their early life (compare dark blue lines between **a**, **b** over ages 1–2 and 3–6). The graphed data refer to the best supported model 22 of Supplementary Table 3. Rank 1 and Rank 2 nestlings had virtually identical survival estimates (differing from the third decimal figure); for clarity, they were thus pooled into a single category to avoid two fully overlapping lines. Based on recapture data from 688 individuals ringed as nestlings. Error bars represent 1 SE. Source data are provided as a Source Data file.

When adult kites encountered a current drought, they suffered on average a 17% reduction in survival (Supplementary Fig. 2, Supplementary Table 3: Model 13). However, taking into account natal conditions (natal drought and brood rank) revealed a more complex effect of drought on survival (Fig. 4, Supplementary Table 3: Model 22). In particular, natal conditions modulated the response to current drought in three ways (Fig. 4). First, natal drought completely overrode the impact of contemporary drought and dictated the survival of all breeding adults, implying a delayed effect still visible decades after birth (during 7–30 years of age; right-most portion of panels a and b of Fig. 4). Second, for most individuals (except Rank 1 nestlings born in normal years and during a current drought, see below), natal drought again imposed costs that overrode those of contemporary drought at all ages (orange vs light-blue lines in Fig. 4). Third, Rank 1 nestlings born in normal years markedly suffered more than others the effect of contemporary drought in their early life (1–2 and especially 3–6 years old; compare dark blue lines between panel a and b of Fig. 4), Thus, these were the only individuals that underperformed when contemporary conditions did not match their natal conditions (compatible with the PAR hypothesis). However, the fact that they underperformed compared to others, but mainly when challenged by drought in younger ages, suggests the action of viability selection rather than PAR mechanisms^28^. While all other individuals suffered a selective filter directly in the nest (by being born with drought or as Rank 2 nestlings that survived the dominance of their older siblings), Rank 1 individuals passed their first mortality bottleneck later, when they encountered a drought in early life. Thus, they enjoyed the benefits of favourable natal conditions in adult life, but only if they passed the filter of their first early life challenge from drought^16,28^. In this sense, drought set individuals on different life history trajectories in a state-dependent manner, based on a social component of developmental conditions, i.e. brood rank^27^.

Overall, our results lend support to the developmental constraint, silver spoon hypothesis, because individuals born during drought were disadvantaged throughout life and enjoyed no benefit when they encountered drought again as adults (Fig. 1 and Supplementary Fig. 3). The potential of adverse developmental conditions to affect aspects of individual morphology, metabolism, immunocompetence, physiology, sociality and personality, with lifelong repercussions, has been demonstrated in the wild as well as in the laboratory^12,19,20,29–31^. In our population, an extreme climatic event experienced at birth through a short period of a few months imposed a disproportionate carry over effect visible throughout life for decades to come. From the individual point of view, the fitness consequences of such natal effects were severe. For example, even assuming an ideal scenario in which a kite never encountered drought as an adult, its average life expectancy would be 9.6 years if born in a normal year, but only 1.2 years if born during a drought, which is insufficient time to produce any progeny.

### Drought impact on population dynamics

**Fig. 5:**
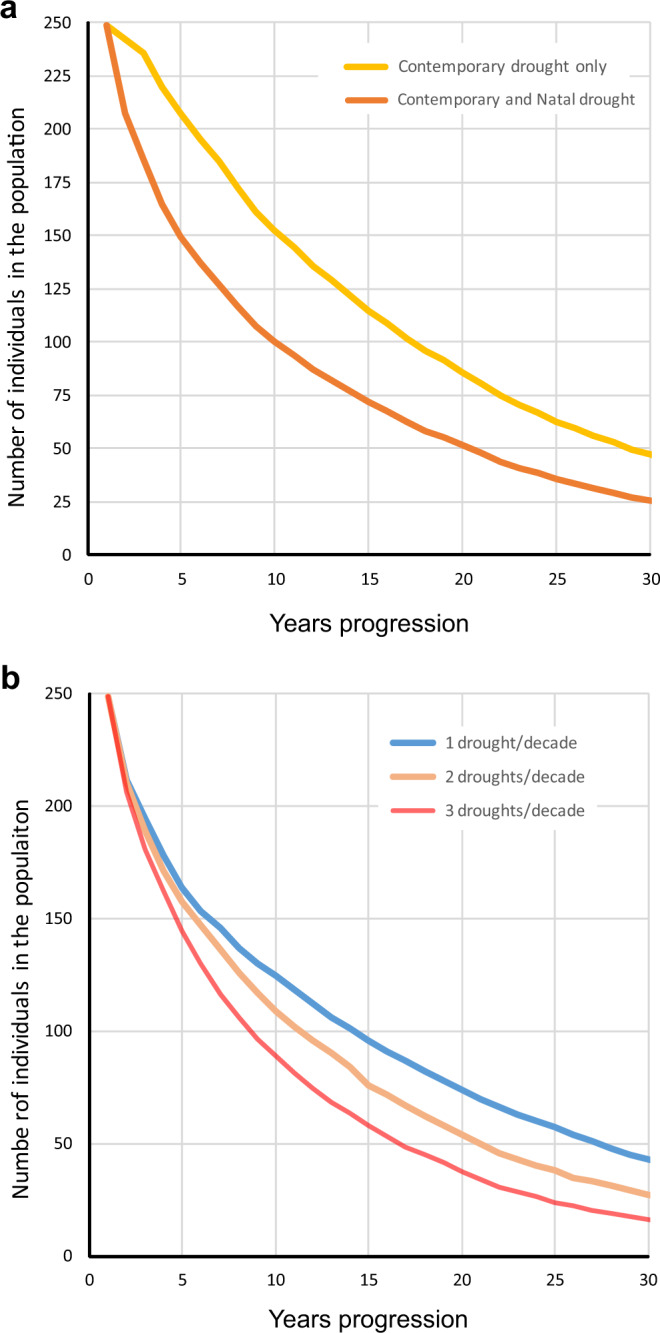
Impact of drought on the population dynamics of red kites in Doñana National Park (southwest Spain). Projected trajectory of the red kite population of Doñana, as estimated by matrix modelling (see Methods), when incorporating (brown line) or discounting (yellow line) the effect of natal drought (**a**). The frequency of drought was set to two per decade. Natal drought, which is usually ignored in assessments of climate impacts, produced a steeper population decline (**a**). An increase in the decadal frequency of drought steepened the projected decline of the population (**b**). Each line is the population trajectory averaged over 100 time-series of randomly occurring droughts (see Methods).

The above effects of drought on all vital rates unavoidably cascaded into population impacts. In Fig. 5 we show the temporal dynamics of this population, as estimated by matrix modelling, under different scenarios of drought frequency (1, 2 and 3 per decade) and with and without the incorporation of natal effects. Two results stand out as important. First, incorporation of the natal effects of drought on the population projected over time substantially speeds up the decline compared to models that only take into account contemporary but not natal drought (the approach commonly employed in assessments of climate impacts) (Fig. 5a). For example, if we discount the impact of natal conditions, an initial population of 249 individuals (rationale in Methods) declined to 152, 86 and 48 individuals after 10, 20 and 30 years, respectively, compared to 100, 52 and 26 individuals when natal effects were included. Thus, incorporating natal effects caused a 34–46% reduction in projected population sizes (mean = 40%). Similarly, if we classify the population as quasi-extinct when only five individuals remain, the time to extinction is 68 years if we discount natal effects but 54 years when including them, i.e. the population goes extinct 14 years earlier. This 21% shortening of extinction latency could mark a large difference in terms of time for conservation interventions by managers^32^. Second, an increased frequency of drought will precipitate faster declines and extinction probabilities (Fig. 5b). When we simulate a stochastic, increasing frequency of one, two and three droughts/decade, the population respectively declined to 125, 109 and 89 individuals after 10 years, to 74, 54 and 38 individuals after 20 years, respectively, and to 43, 27 and 16 individuals after 30 years. Thus, as drought frequency increased from one to two to three events/decade, the average population decline increased from 68% to 75% to 81%, while the time to extinction declined from 73 to 54 to 44 years.

Thus, drought-effects undermined all components of demographic performance, including the survival of breeding adults, which often is buffered from environmental variation, and a major driver of population dynamics in long-lived species^33–35^. Such depression of vital rates can generate an intermittent, demographic bottleneck, whose long-term population impact is determined by the frequency and severity of drought^36–38^. However, the mechanisms behind such impacts in red kites were more complex than currently appreciated, and acted through two pathways: a direct lowering of adult vital rates, as well as a reduction of the fitness of progeny born during drought, which injected a pool of weak performers into the population that acted as a long-term form of carry-over effect on population dynamics, i.e. a climate legacy. However, this carry-over was more subtle than a simple cohort-effect, because its impact was modulated through time by another natal effect (brood rank) and by the frequency of hardship encountered again in early life (Fig. 4). The combined action of a direct impact in the present and a diffuse weakening of individuals in the future intensified population declines, lowered projected abundance and accelerated the time to potential extinction. This process could have severe consequences, especially for populations that are already small and declining, such as those of many endangered species, which would not be able to absorb additional pressures^39,40^.

These results have important conservation implications. First, whenever climate or global change impose substantial natal effects, population declines will likely be faster and steeper than currently appreciated. Second, the natal effects of climate change may be subtle and difficult to link to their drivers in standard population models that simply relate population properties to simultaneous climate conditions. Furthermore, these natal effects could weaken and erode populations for years in a cryptic manner, resulting in sudden crashes that appear difficult to explain. This could contribute to the mysterious large-scale declines recently documented for many taxa^41–44^. Third, the protection of long-lived species often focuses on adult survival as the main target of management action, given its disproportionate role in driving population growth^33–35^. Management targeting breeding success is often viewed as inefficient and mainly implemented under emergency conditions, e.g. when natality rates are unsustainably low^45^. However, when adult survival is jeopardised by natal conditions, as shown here, offspring-based management may entail much broader benefits than a simple improvement in fecundity. For example, in our kite population, a strategic program of supplementary feeding of offspring during drought could buffer the population from the natal effects of climate extremes and simultaneously alleviate sibling rivalry^45^. All this would require management to refocus on natal conditions in order to improve the long-term adult performance. Recognizing and manipulating the pathways that link natal conditions to adult performance and population dynamics could yield substantial management benefits, considering that the full buffering of natal effects would have increased kite population size by an average 40% in our simulations.

In conclusion, extreme climatic events and the ecological disturbances that they trigger will likely become more frequent and intense in the future, with far-reaching impacts on individuals and populations of many species. We have shown that the mechanisms behind such impacts may be more complex and protracted than previously appreciated, potentially carrying over for decades and becoming apparent when it may be too late. In particular, acknowledging and addressing the actions of natal effects and climate legacies can radically alter the severity of population declines and shorten the time to extinction. Similar synergies between natal and current conditions may apply to any type of anthropogenic impact provoking stress and hardship for both offspring and adults (e.g. chemical contamination, human disturbance, etc.). Of course, recognition of the relevance of natal effects does not imply that current effects should be viewed as less important. For example, should a major detrimental factor suddenly emerge, it could override other threats, including natal effects, if it strongly impacts a life history stage that is key for population growth. Overall, our results imply that climate and global change may be eroding populations more quickly and severely than generally recognized. Thus, there is an urgent need to incorporate potential “penalties” for natal effects in current forecasts of climate impacts as conservative, worst-case scenarios. For some species, such penalties could be substantial, as shown here. Incorporating natal effects into the analytical toolkit of climate and global change biology will make future forecasts more realistic and may contribute to resolve the enigma of many unexplained large-scale declines and extinctions.

## Methods

This study was conducted in accordance with the national and European laws concerning the use of animals for scientific purposes (protocols EBD-11/09 and EBD-11/25 approved by the Animal Care and Bio-Ethics Sub-Committee of the Consejo Superior de Investigaciones Científicas - CSIC).

### Study area

Doñana National Park is located in the estuary of the River Guadalquivir, along the coast of the Atlantic Ocean in south-western Spain. In a typical, “normal” year, autumn-winter rainfall causes extensive flooding of vast expanses of land (Fig. 2). The extent of such seasonal marshland peaks in February-March and then surface water progressively declines due to increasing spring-summer evapotranspiration, so that little water is left by June-July^46^. In exceptional drought years, however, autumn-winter rainfall is extremely scarce and the marshes flood little if at all. By February-March in a drought year, the majority of the park appears as brown-to-grey dry soil with scarce to barren vegetation, in stark contrast with the luxuriant, green-blue looking ecosystem of normal years (Fig. 2). In recent times, climatological droughts have been amplified by growing levels of unsustainable and often illegal water extraction for agricultural purposes in non-protected lands bordering the park^47–50^. This is causing new types of drought, such as those of the years 2014 and 2016 when the marshes became inundated unusually late and then dried much faster than usual, functionally acting as drought for most predators and prey in the ecosystem. Furthermore, climate models forecast a substantial, future increase in drought frequency and severity for the region (Andalusian region and southern Spain)^51–53^.

In this analysis, we classified drought conditions as years in which inundation levels were below the 20th percentile of maximum marsh flooding on 30 March (the average kite laying date) in the historical record of all the available cloud-free Landsat MSS, TM and ETM + scenes for the Doñana region during the period 1975–2018 (528 images). Images were radiometrically corrected, transformed into reflectance values and normalized to a reference image to produce final inundation masks based on pixels of 30 m x 30 m (e.g. ref. 54; examples in Fig. 2c, d). So defined, drought implied the flooding of less than a quarter of the marsh bed. These conditions occurred in 10 of the 44-year data series (1981, 1983, 1993, 1994, 1995, 1999, 2005, 2012, 2014 and 2016), corresponding to an average frequency of two drought years per decade. The impact of drought intensity and duration will be analysed elsewhere.

### Model species

The red kite (hereafter “kite”) is a long-lived raptor (maximum lifespan of 30 years) virtually endemic to Europe. Upon birth, kite eggs hatch asynchronously, imposing a size-based dominance hierarchy among nestlings, where the first-born (“Rank 1 nestling”) enjoys a feeding and growth advantage over its subdominant second- and third-born siblings (“Rank 2 nestlings”). The disadvantages suffered by Rank 2 nestlings include diminished access to food, and stress and injuries from physical attacks by their larger, dominant sibling^55,56^. Kites start to breed between one and seven years of age, usually at age three, so that all individuals are breeding by age seven onwards^57,58^. As a result, the life cycle revolves around three major, sequential life stages: (i) predominantly non-breeding individuals in their initial two years of life (hereafter “juveniles”); (ii) 3–6 years old “young adults” attempting to recruit or in their initial, frequently unsuccessful breeding attempts; and (iii) “breeding adults” above seven years of age with maximum survival and breeding performance^59^. In Spain, kites are year-round residents and declining over vast portions of their range, including Doñana and its surrounding region, where they are classified as critically endangered^60^. In Doñana, kites preferentially breed close to the seasonal marshes, subsisting on a varied diet dominated by aquatic birds, fishes and other prey that are mainly concentrated in or close to the marshes^55,61^. Therefore, the red kite can be considered one of the main top predators of the marshland ecosystem and depends highly on it for food. This makes the red kite a potential sentinel of the ecosystem-wide impact of drought and a good model organism to investigate the mechanisms by which a climate extreme could affect fitness, undermine population viability and drive the decline of an endangered species.

### Field procedures

Red kites have been surveyed since the 1970s (for details of survey and nest check procedures, see ref. 60). Whenever possible, nests were checked multiple times in order to ascertain: whether laying took place, the number of eggs laid, their hatching success, nestling mortality, and the number of chicks raised to fledging age per territorial pair (a pair that holds a territory), per breeding pair (one that lays eggs) and per successful pair (a pair that raises at least one nestling to fledging age). A nest was classified as failed by predation when we found remains of the nestlings plucked and consumed in or under the nest, or when predation was documented by camera-trapping (see below).

Since 1986, the nestlings were marked with a white Darvic ring with a black three-character alphanumeric code, which can be read from a distance with a spotting scope. Upon ringing, for each nestling we measured body mass to the nearest 5 g, tarsus length to the nearest 0.1 mm, and the length of the third primary to the nearest 1.0 mm. For survival models, we analyse 190 reencounters of 688 kites ringed as nestlings in Doñana National Park between 1986–2018. As a consequence, we know the exact chronological age of all birds in the dataset. Encounters included birds re-sighted on their territories or on carrion-baits through 2019 (details in ref. 59).

To test whether drought affected annual food availability, we surveyed prey abundance transects during the kite nestling stage at 30 locations randomly chosen within the kite study area during two years of drought (2012 and 2014) and a preceding or consecutive normal year (2011 and 2015). For each transect, the observer: (1) reached the site through a hand-held GPS; (2) waited 10 min for the local fauna to resume activities; and (3) slowly walked a 200 m transect, annotating all potential prey species heard or seen within a 100 m distance. Prey included all species that appeared at least once in kites’ diet, based on more than 6500 prey remains identified, and any other species reputed as potential prey, based on our general knowledge of kite hunting methods. To take into account differences in prey mass, each prey individual was converted to its mass and all masses were summed for each location to obtain an overall index of cumulative biomass of available prey. The main objective of these transects was to obtain a rapid snapshot of overall prey availability for a very generalistic raptor over a large area, in order to give an idea of its variation between drought vs non-drought years. Unavoidably, such transects did not adequately sample some prey categories that could be important for kites, such as fishes, small mammals or carrion. However, (1) incorporating fish availability would have obviously caused a greater decline in prey availability during drought, which makes our prey assessment more conservative; (2) small mammals are a rare prey for kites in Doñana (<1 % of the diet by mass); and (3) carrion, which is mainly consumed by kites in the winter, was probably more available during drought due to increased herbivore mortality, but its temporary contribution to diet would be highly unlikely to change the decline in prey availability caused by drought.

Finally, to examine whether drought affected the capability of parents to provision their nestlings, we placed camera-traps within 1–2 m of kite nests. Cameras incorporated a motion-sensor that triggered photographs every time it detected movement in the nest, thus capturing images of prey deliveries. For this analysis, we only included: (1) days in which all prey items were identified to species level, to assign a body mass to each prey item; and (2) years in which we could sample at least 10 full days of prey deliveries from a minimum of five nests. This yielded a sample of 97 full provisioning days from 39 nests from three drought years (2012, 2014, 2016) and three normal years (2013, 2017, 2018). For each provisioning day, we calculated the cumulative mass of prey delivered to a nest and divided it by brood size to obtain an estimate of daily provisioning rate per nestling. Being at the daily scale, these data are poorly suited to investigate changes in dietary diversity, which will be examined in the future.

### Statistical analyses

To investigate whether drought affected nestling body size and condition, we: (1) estimated the age of a nestling (see equation below); (2) calculated its theoretical body size and mass for that age (see equations below); and (3) subtracted the expected value from the observed value (i.e. the measurement at ringing). Thus, for example, two nestlings that weighed 800 and 900 g at 50 days of age, when their expected mass should be 870 g, had a negative residual (mass deficit) of −70 g and a positive residual (mass gain) of 30 g, respectively, as estimates of their body condition. Body size was estimated by the length of the tarsus (the only available estimate of skeletal size throughout the study). The relationship between age and body measures was evaluated by building growth curves through linear mixed effects models, following the procedures outlined by^62^, imposing a logistic curve for body mass and tarsus length and a linear progression for the length of the third primary feather^55^. For these models, we used data from an intensive study of this population’s growth curves, based on 491 measurements from 64 nestlings whose birth date was exactly known^55^. Based on these models, nestling age was backdated from feather development with the equation: age = 0.139 * (3rd primary length) + 17.251 (R^2^ = 0.97), while expected body mass and tarsus length were estimated with the equations: mass = 879.453/(1 + 2.829^((22.479 - age) * 0.160)) and tarsus length=56.585/(1 + 2.829^((11.639 - age) * 0.113)). The best supported random structure for these three equations was: (1) a random effect of nestling identity for feather length; (2) two random effects of nestling identity nested within brood identity for asymptotic mass and for the inflection point of the mass logistic curve; (3) two random effects of nestling identity nested within brood identity for the tarsus growth rate constant and for the inflection point of the tarsus logistic curve^62^.

To examine the impact of drought on prey availability, on offspring provisioning rates, on components of breeding performance (e.g. clutch size, number of fledged young, etc) and on nestlings body size and condition, we built a series of linear mixed models and generalized mixed models, with drought as the explanatory variable, with an error, link and random structure detailed in Supplementary Table 1. For models of body size and condition, we also fit brood rank and its interaction with drought as additional explanatory variables. An explanatory variable was considered important when it caused a significant increase in model deviance as determined by a Likelihood Ratio Test and an increase of more than 2 AICc-units upon its removal from the global model^63^. Continuous variables were standardized before being fitted to mixed models.

### Survival modelling

Survival probabilities were estimated from observations collected from 1986 to 2019 (number of occasions=34) of 688 kites marked as nestlings. Observations were conducted each year during the breeding period and coded into individual encounter histories. Animals were sorted into four groups according to their birth rank (two levels: first born *vs* second and third born) and the condition of the marshland in the year of birth (two levels: drought *vs* normal natal conditions). Survival (ϕ) and detection probabilities (p) were modelled and estimated simultaneously through capture-mark-recapture models implemented in MARK 9.0^64^. Informative goodness-of-fit tests for capture-mark-recapture models are only available for a general model in which recapture probability and survival are both time- and group-dependent. The test identified a difference between newly and already marked birds, an indication of an age difference in survival (χ^2^_34_ = 68.64, *P* < 0.001). The presence of an age effect was expected because survival progressively increases in this population, with age groupings from 1–2, 3–6, and 7–30 years old^59^. Here, we considered the same age structure, but a model assuming survival and detection parameters dependent on age, time and their statistical interaction would have been over-parametrized. As a consequence, we began model selection from a simpler model assuming no time effect but an effect of age class, brood rank, natal conditions and their statistical interactions on survival and detection probabilities. The goodness-of-fit of this model was assessed using the bootstrap method^64^ with 1000 simulations (*P* = 0.307). We reduced the year-effect to a two-level factor by considering years in which the marshland was flooded and years in which it was dry (normal *vs* drought years, hereafter ‘contemporary conditions’). Model selection was based on AIC. Maximum likelihood parameter estimates were obtained from the best supported model (i.e. with the lowest AICc value^65^) using program MARK 9.0^66^. The rationale behind model selection was based on testing the hypothesized mechanisms at play (e.g. see Introduction and Fig. 1), namely the impact of natal conditions (marshland inundation in natal year and brood rank), and of contemporary conditions encountered in any given year on both survival and detection probabilities (Supplementary Table 2). We built our models gradually towards the most complex structure, by first assessing the importance of natal and contemporary conditions separately, and subsequently implementing a consensus model that incorporated the parameter structure retained from each of these two earlier independent selections. Once we reached this consensus model, we re-assessed the significance of those effects and interactions that had been dropped in the earlier stages of the analysis (see also ref. 67).

Below, we briefly outline the methodological steps that led to the final consensus model, so as to better focus the reader on the relevant graphical implications of the survival-models in the Results section. The first two independent steps of our model selection aimed at assessing the effect of natal and contemporary conditions. They led to a consensus model in which detection varied according to age and contemporary drought. The interaction term between these two effects was not retained (Supplementary Table 3). An additive effect of age and contemporary drought was also retained for survival, together with natal drought and Rank. In the third and final step, we further simplified the model by re-assessing the effects dropped in the earlier steps of the analysis. The retained model (Model 22 Supplementary Table 3) had an additive effect of age, Rank and contemporary drought. Note that the detection probabilities of kites born in normal years were similar to the detection probabilities of kites born during drought. Although a model that included an effect of natal drought had a similar AICc value (Model 23), this effect played the role of a pretending variable (identified by the lack of reduction in the deviance, indicating the model fit did not improve; see ref. 65: page 65–66) and the model was thus not considered any further. According to the final model (Model 22), survival probability varied by age, by natal conditions and by contemporary conditions. This model assumed an additive effect of all terms. However, first-born chicks had a different pattern of survival according to age, i.e. an interaction term between age and rank.

### Population projections and modelling

To explore the effect of drought on population trajectories, we built a post-breeding age-structured matrix population model, as in ref. 59 (Supplementary Code 1). This model contained the age-dependent fecundity and survival probabilities (for eight ages) to project the population state from time *t* to *t* + *1*^68^ and it is also referred to as a “transition matrix”. The survival parameters were calculated pooling Rank 1 and Rank 2 individuals and thus weighted the relative contribution of both type of siblings. To investigate the interplay of natal conditions and contemporary conditions (Fig. 1), we started by considering two groups of birds according to the environmental conditions at birth (natal drought vs normal year, respectively). The dynamics of these two sub-groups of the population was investigated along a stochastically simulated time-series of wet (normal) and drought conditions, i.e. using a random sequence of contemporary conditions. At a given time interval from *t* to *t* + *1*, the transition matrix was calculated according to the set of parameters imposed by the conditions encountered (Supplementary Table 4; see refs. 38,69 for similar approaches). For example, during a drought event, individuals in both groups of Supplementary Table 4 survive and breed according to the parameters estimated for contemporary drought and all chicks produced are classed as born under a natal drought (all parameters specified in Supplementary Table 4). On the contrary, in normal years, kites survive and breed according to the parameters typical of non-drought contemporary conditions and all chicks produced are classed as born under normal natal conditions (Supplementary Table 4). We built four transition matrices corresponding to the 2×2 combinations of natal and contemporary droughts and calculated the respective population growth rates (λ). For each transition matrix, we estimated the uncertainty of λ through simulations (*N* = 1000) that took into account the variability of each parameter, as estimated by its standard error (Supplementary Table 4). To explore the impact of drought and the relative role of natal vs contemporary conditions, we built 100 time-series of contemporary conditions in which drought events occurred at random with frequency *z*, where *z* = 0.1, 0.2 and 0.3 (i.e. 1, 2 and 3 droughts per decade, on average). We then used such sequence of wet and drought events as a driver of simulated population trajectories (see ref. 38 for a similar approach). For each scenario of drought frequency, we simulated 100 trajectories and then calculated the average population size at each point in time. We started all simulations form an initial population size of 249 individuals. This figure would represent the total Doñana population of breeders and non-breeders estimated for the 1990s^59,60^ and was considered as a biologically realistic starting point to illustrate changes in population trajectory according to natal and contemporary drought. The R code for all the population-modelling above can be found in Supplementary Code 1.

### Reporting summary

Further information on research design is available in the Nature Research Reporting Summary linked to this article.

## Supplementary information

Supplementary Information

Description of Additional Supplementary Files

Supplementary Code 1

Reporting Summary

## Data Availability

The data generated in this study are available from Digital.CSIC at 10.20350/digitalCSIC/14705. Source data are provided with this paper.

## Source data

Source Data

## Code availability

The R code for the population simulations is available in Supplementary Code 1.
