# Supplementary material for: Hardship at birth alters the impact of climate change on a long-lived predator
Source: Nat Commun. 2022 Sep 27;13:5517. doi: 10.1038/s41467-022-33011-7 (PMC9515099; doi:10.1038/s41467-022-33011-7)
Supplement: Supplementary file 1 — Supplementary Information [file 41467_2022_33011_MOESM1_ESM.pdf]

Supplementary Information

Hardship at birth alters the impact of climate change on a long-lived predator

Sergio, F., Tavecchia, G., Blas, J., Tanferna, A., Hiraldo, F., Korpimaki, E. and Beissinger, S.R.

Table of contents:

- Supplementary Fig. 1
- Supplementary Fig. 2
- Supplementary Fig. 3

- Supplementary Table 1
- Supplementary Table 2
- Supplementary Table 3
- Supplementary Table 4

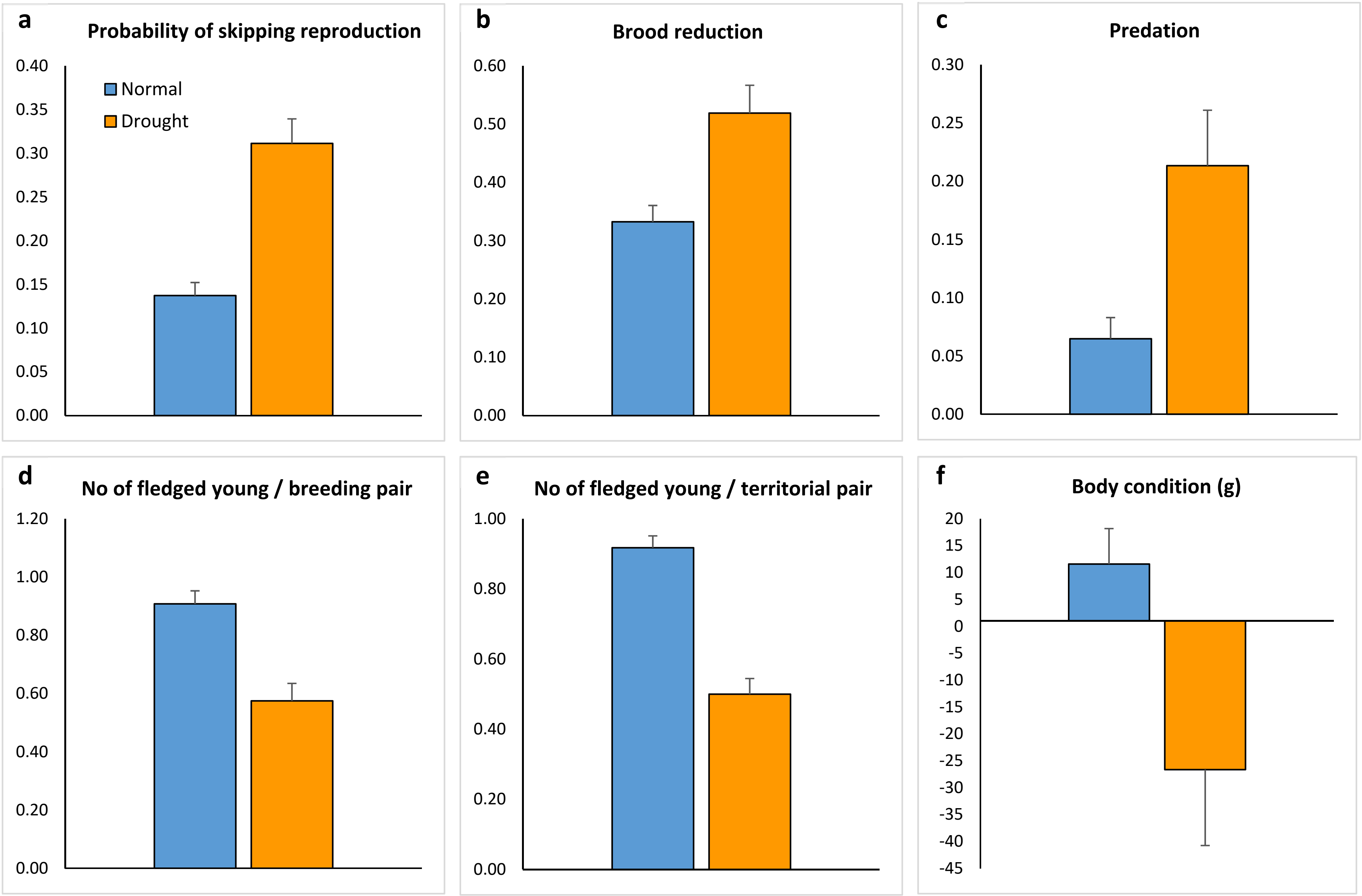

Supplementary Fig. 1. Drought increased the probability of skipping reproduction (i.e. not laying eggs, panel a, N = 532 and 273 breeding attempts for normal and drought years, respectively), the mortality rate of hatched nestlings (brood reduction, panel b, N = 183 and 79) and the rate of nest predation (c; N = 185 and 75), thus depressing the number of fledged young (panel d and e; N = 455 and 181; and N = 840 and 316), as well as their body condition (panel f; mass deficit: observed mass of a nestling compared to the mass expected for its age, see Methods; N = 273 and 94). Data are presented as mean values ± 1 SE. Source data are provided as a Source Data file.

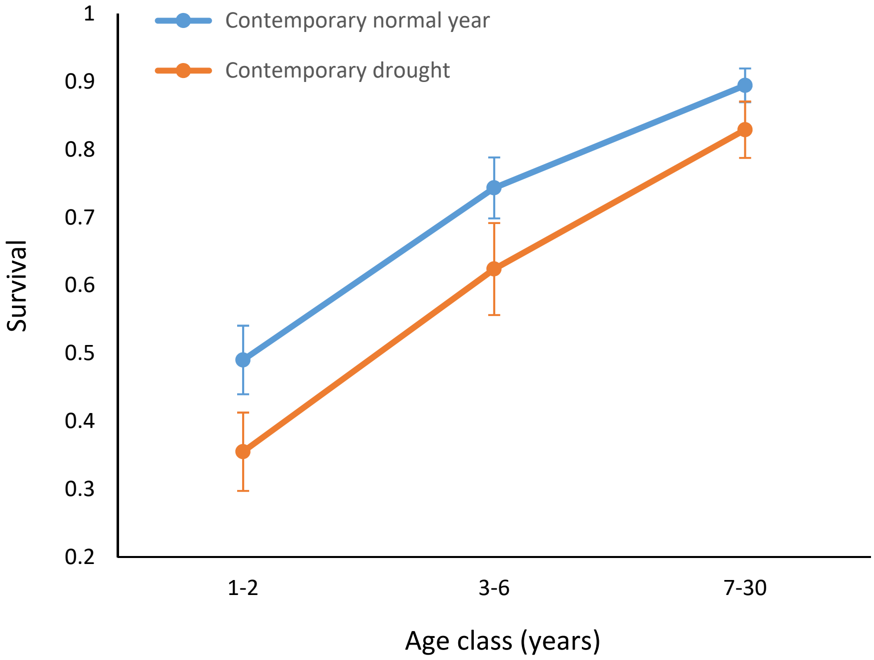

Supplementary Fig. 2. Lifelong, age-dependent survival of the red kites of Doñana National Park (southwest Spain) when they encounter a drought (orange line) or a year of normal marsh inundation (blue line). The data portrayed in this graph are based on model 13 of Supplementary Table 3. Error bars represent 1 S.E. Based on recapture data from 688 individuals ringed as nestlings. Source data are provided as a Source Data file.

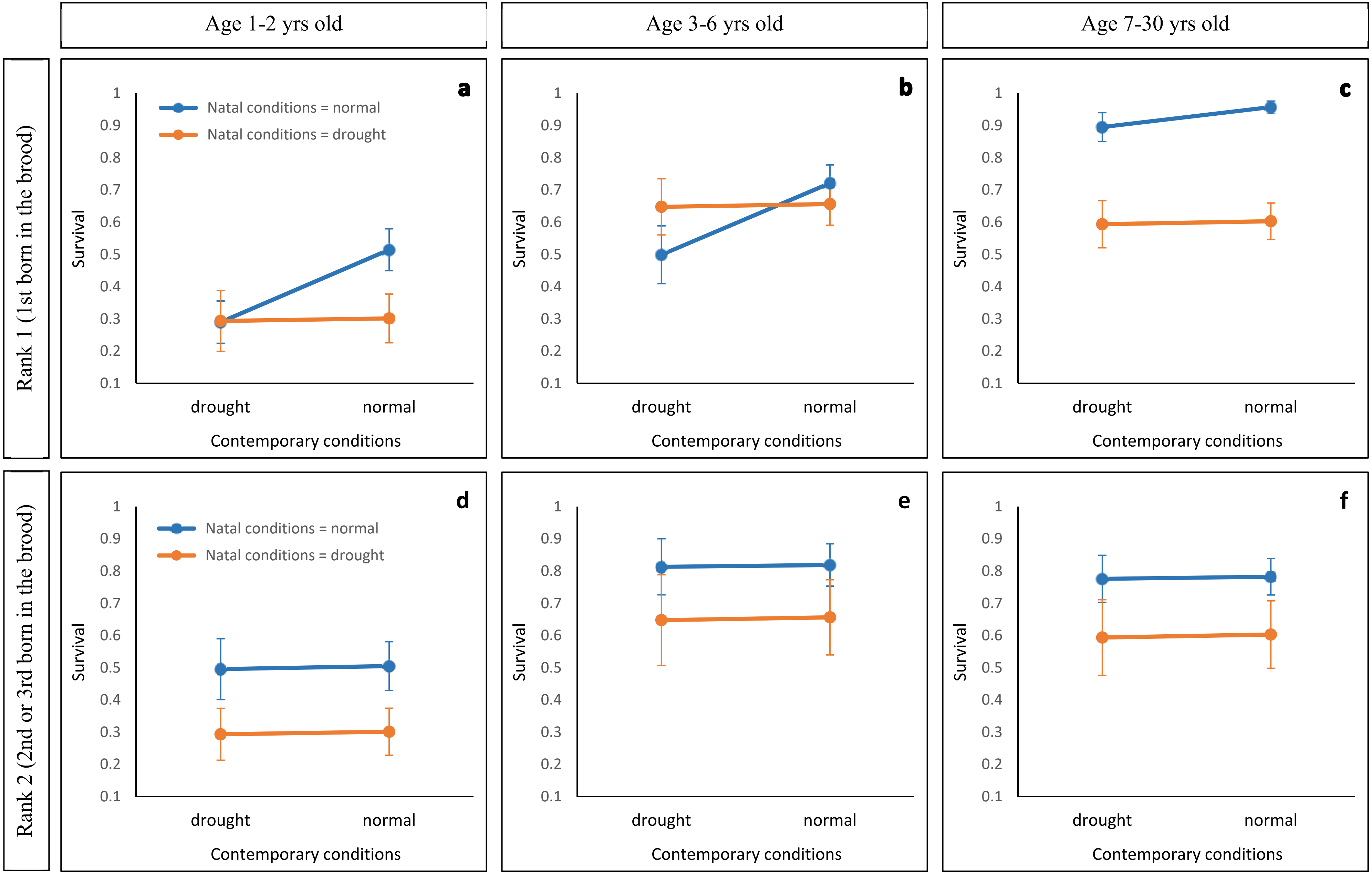

Supplementary Fig. 3. Variation in survival as a function of conditions during development (natal conditions) and in later life (contemporary conditions) for individuals of different brood Rank (1st born in the brood: panels a, b, c vs 2nd and 3rd born in the brood: panels d, e, f) and at different stages of life (age 1-2: panels a and d; age 3-6: panels b and e; age 7-30: panels c and f). Conditions ranged from the hardship of drought to the favourability of years of normal marsh inundation. Under most circumstances, the lines ran parallel to each other, in support of the "developmental constraint", or "silver spoon" hypothesis, where individuals born under hardship are disfavoured for the rest of their life, independently of adult conditions (see predictions in Fig. 1). The crossing

lines of panel b, which could support the "predictive adaptive response" hypothesis (Fig. 1), are more likely to be driven by viability selection (see Discussion). Error bars represent 1 S.E. Based on recapture data from 688 individuals ringed as nestlings. Source data are provided as a Source Data file.

Supplementary Table 1. Mixed models testing the effect of drought on prey availability, parental provisioning rates to their offspring, components of breeding performance, and the body size and condition of nestlings of the red kite population of Doñana National Park (southwestern Spain). All tests are two-tailed.

Variable (sample size):	Parameter estimate ± SE	LR test [a]	P	ΔAIC [b]
**a. Dependent variable: Prey availability (120) [c]**				
Drought [g]	0.86 ± 0.17	12.60	0.0004	10.42
Intercept	-0.43 ± 0.05	-	-	-
**b. Dependent variable: Offspring provisioning rates (97) [d]**				
Drought [g]	1.03 ± 0.26	10.97	0.0009	8.74
Intercept	-0.42 ± 0.18	-	-	-
**c. Dependent variable: Probability of breeding (805) [e,f]**				
Drought [g]	0.97 ± 0.22	19.97	< 0.0001	17.95
Intercept	1.89 ± 0.79	-	-	-
**d. Dependent variable: Clutch size (497) [h]**				
Drought [g]	0.03 ± 0.07	0.28	0.60	-1.76
Intercept	0.80 ± 0.06	-	-	-
**e. Dependent variable: Hatching success (272) [i]**				
Drought [g]	0.13 ± 0.10	1.91	0.17	-0.14
Intercept	-2.20 ± 0.08	-	-	-
**f. Dependent variable: Brood reduction (260) [j]**				
Drought [g]	0.39 ± 0.13	9.20	0.002	7.14
Intercept	-2.20 ± 0.08	-	-	-
**g. Dependent variable: Predation rate (260) [e,k]**				
Drought [g]	1.35 ± 0.77	8.29	0.004	6.23
Intercept	2.06 ± 0.82	-	-	-
**h. Dependent variable: Fledged young/breeding pair (637) [h,l]**				
Drought [g]	0.39 ± 0.11	12.4	0.0004	10.35
Intercept	-0.55 ± 0.08	-	-	-
**i. Dependent variable: Fledged young/territorial pair (1158) [h,m]**				
Drought [g]	0.49 ± 0.09	28.5	< 0.0001	26.46
Intercept	-2.20 ± 0.08	-	-	-
**j. Dependent variable: Body condition (367) [n]**				
Drought [g]	0.27 ± 0.13	4.52	0.035	2.44
Brood Rank	-0.72 ± 0.09	54.73	< 0.001	52.65
Intercept	0.02 ± 0.11	-	-	-
**k. Dependent variable: Body size (354) [n]**				
Drought [g]	0.12 ± 0.13	0.86	0.354	1.22
Brood Rank	-0.66 ± 0.06	82.76	< 0.0001	80.68
Intercept	0.07 ± 0.12	-	-	-

[a] Likelihood ratio test of the change in deviance upon removal of the variable from the model.

[b] ΔAIC is the increment in AIC that would derive by the removal of the variable from the model.

[c] Linear mixed model with normal errors and an identity link. Year was fitted as a random term. The model included a VarIdent correlation structure to take into account the lower variation in prey availability under drought conditions.

[d] Linear mixed model with normal errors and an identity link. The sample unit is the biomass of prey/nestling delivered by the parents in a full day of camera-trapping at the nest. Identity of nesting territory and year were fitted as random effects.

[e] Generalised linear mixed model with binomial errors and a logit link function. Identity of nesting territory and decade were fitted as random effects.

[f] Probability of breeding vs skipping reproduction (0 = the female of a pair never laid eggs and skipped reproduction; 1 = the female laid eggs).

[g] Categorical variable with two levels: 1 = drought; 2 = normal year.

[h] Generalised linear mixed model with Poisson errors and a logarithmic link function. Identity of nesting territory and decade were fitted as random effects.

[i] linear mixed model with Poisson errors and a logarithmic link function. Hatching success was the percentage of eggs that hatched and was examined by fitting the number of hatched nestlings as the dependent variable and the number of laid eggs as an offset [1]. Identity of nesting territory and decade were fitted as random effects.

[j] linear mixed model with Poisson errors and a logarithmic link function. Brood reduction was the percentage of hatched nestlings that died before fledging and was modelled by fitting the number of fledged nestlings as the dependent variable and the number of hatched nestlings as an offset [1]. Identity of nesting territory and decade were fitted as random effects.

[k] Categorical variable with two levels: 0 = nest predated; 1 = nest not predated.

[l] Number of nestlings raised to fledging age per breeding pair (i.e. a pair that laid eggs).

[m] Number of nestlings raised to fledging age per territorial pair (i.e. a pair that held a territory and laid eggs or not).

[n] Linear mixed model with normal errors and an identity link. The sample unit was an individual nestling. Brood rank was the order of birth in the brood (1 = firstborn nestling; 2 = second or third born sibling; there were too few third-born nestlings to be analysed as a separate level). Body condition and size were estimated as the difference between the observed and expected mass and tarsus length of a nestling, given its age (see Methods). Explanatory variables fitted to the model were: drought, brood rank and drought*brood rank. Brood identity and decade were fitted as random terms. The model included a VarIdent correlation structure to take into account the lower variation in body size and condition by firstborn siblings.

Supplementary Table 2. Effects tested in survival models and their model notation. The symbol "_" denotes the range of ages included in an age class (e.g. 3_6 implies an age class that includes individuals of three to six years old).

**Effect considered**	**Levels**	**Effect**	**Model notation**
Age	3	1_2 / 3_6 / 7_30	age
Contemporary drought	2	Normal *vs* Drought	contemporaryD
Rank	2	1 / 2_3	rank
Natal drought	2	Normal *vs* Drought	natalD
Statistical interaction between main effects	-	-	*
Additive relationship between main effects	-	-	+

Supplementary Table 3. Survival probability (ϕ*)* and detection probability (p) as a function of natal drought (noted 'natalD'), brood Rank (noted 'rank') and contemporary drought (noted 'contemporaryD'). Model selection proceeded in three steps. First, we modelled natal drought and brood Rank (i.e. two types of early, natal effects). Second, we modelled the effect of contemporary drought. Third, we built a consensus model by considering the effects retained in the previous steps (see details in Methods). All models assumed a 3-levels ageclass structure in survival, based on previous work on the same population [2]. Np = number of estimable parameters in the model. The most plausible model retained in each selection step is highlighted in bold.

Model	Survival (ϕ)	Recapture (p)	np	Dev	AICc	ΔAICc
Early conditions and rank						
**9**	**age*rank*natalD**	**age**	**12**	**717.062**	**1346.045**	0.00
8	age*rank*natalD	age	14	714.301	1347.410	1.37
7	age*rank	age*rank	12	720.753	1349.736	3.69
1	age* rank*natalD	age*rank*natalD	21	703.418	1351.123	5.08
4	age*rank	age	9	728.438	1351.267	5.22
5	age*natalD	age	9	730.522	1353.351	7.31
6	age	age*rank	9	732.072	1354.900	8.86
3	age*natalD	age*natalD	12	727.205	1356.187	10.14
2	age	age*rank*natalD	15	724.011	1359.191	13.15
Contemporary drought						
**13**	**age+contemporaryD**	**age+contemporaryD**	**8**	**715.530**	**1336.317**	**0.00**
14	age	age+contemporaryD	7	719.805	1338.555	2.24
11	age*contemporaryD	age+contemporaryD	10	715.299	1340.174	3.86
10	age* contemporaryD	age*contemporaryD	12	712.939	1341.922	5.61
12	age* contemporaryD	age	9	739.57	1362.402	26.09
Consensus model: early conditions, rank and drought						
**22**	**(age+contemporaryD+natalD)*rank [a]**	**age+rank+contemporaryD**	**15**	**681.962**	**1317.142**	**0**
23	(age+contemporaryD+natalD)*rank [a]	age+rank+ contemporaryD+natalD	16	681.427	1318.682	1.54

19	age*rank* contemporaryD	age+rank+contemporaryD	22	670.566	1320.376	3.234
21	(age+contemporaryD)*rank*natalD	age+rank+contemporaryD	19	677.322	1320.833	3.691
15	age*rank* contemporaryD	age+contemporaryD+rank+natal D	23	670.969	1322.887	5.745
18	age*rank* contemporaryD	age+contemporaryD	21	679.845	1327.55	10.408
13	age*rank* contemporaryD	age*contemporaryD	23	677.21	1329.129	11.987
14	age*rank* contemporaryD	age*contemporaryD*rank*natal D	37	650.808	1332.792	15.65
17	age*rank* contemporaryD	age+rank	21	696.334	1344.039	26.897
20	age*rank* contemporaryD	age+rank+natalD	22	697.577	1347.386	30.244
16	age*rank* contemporaryD	age+natalD	21	707.106	1354.811	37.669

[a] The interaction term is only applied to first-born (Rank 1) nestlings born under normal (wet) conditions.

Supplementary Table 4. Parameters (S.E.) used in the transition matrix associated with natal and contemporary conditions. The breeding proportion (bp_j) for birds of age j (with j=1 to 6+) was 0.05, 0.1, 0.85, 0.9, 0.95 and 1.00, respectively (following Sergio et al. 2021). Full recruitment occurred at 7 years of age (Sergio et al. 2021). The breeding proportion and the number of chick fledged per territorial pair were assumed to be independent from natal conditions but dependent on contemporary conditions. The values are based on observed mean estimates for drought and normal conditions. nF= number of chick fledged per territorial pair, λ = asymptotic population growth rate; "_" = the range of ages included in an age class. The S.E. of λ was calculated by simulating 1000 stochastic trajectories.

	Natal conditions:	*Normal*		*Drought*	
	Contemporary conditions:	*Normal*	*Drought*	*Normal*	*Drought*
Parameter	*Age*				
Survival	1_2	0.521 (0.053)	0.345 (0.061)	0.318 (0.105)	0.273 (0.109)
	3_6	0.762 (0.045)	0.608 (0.071)	0.698 (0.201)	0.650 (0.250)
	7_30	0.905 (0.024)	0.821 (0.044)	0.454 (0.344)	0.400 (0.344)
nF	1_30	0.918 (0.034)	0.500 (0.045)	0.918 (0.034)	0.500 (0.045)
λ		0.969 (0.020)	0.842 (0.039)	0.657 (0.131)	0.567 (0.159)

Supplementary References

1. Zuur, A. F., Ieno, E. N., Walker, N. J., Saveliev, A. A. & Smith, G. M. *Mixed effects models and extensions in ecology with R.* (Springer, 2009).
2. Sergio, F., Tavecchia, G., Blas, J., Tanferna, A. & Hiraldo, F. Demographic modeling to fine-tune conservation targets: importance of pre-adults for the decline of an endangered raptor. *Ecol. Appl.* **31**, 1–12 (2021).
