# Supplementary material for: Hardship at birth alters the impact of climate change on a long-lived predator
Source: Nat Commun. 2022 Sep 27;13:5517. doi: 10.1038/s41467-022-33011-7 (PMC9515099; doi:10.1038/s41467-022-33011-7)
Supplement: Supplementary file 2 — Description of Additional Supplementary Files [file 41467_2022_33011_MOESM2_ESM.pdf]

Description of Additional Supplementary Files

File Name: Supplementary Code 1
Description: Impact of natal and current climate on the population dynamics of a long-lived predator.
