# Supplementary material for: Hardship at birth alters the impact of climate change on a long-lived predator
Source: Nat Commun. 2022 Sep 27;13:5517. doi: 10.1038/s41467-022-33011-7 (PMC9515099; doi:10.1038/s41467-022-33011-7)
Supplement: Supplementary file 3 — Supplementary Code 1 [file 41467_2022_33011_MOESM3_ESM.zip › ReadMe.docx]

The ZIP file contains several R-scripts to run the analysis in program “R”. They are used sequentially.

They make use of libraries that can be install in program R using these commands.

install.packages('popbio')

install.packages('plot3D')

install.packages('matrixStats')

install.packages("devtools")

install.packages("truncorm")

Here below a brief description of each script.

1) **source("1_MR_2021_PARAM.R")** : Builds 4 matrices N1, N2. N3, N4 corresponding to natal conditions (N1 and N3) and contemporary conditions (N2 and N4). This is a deterministic model.

2) **source("1_MR_2021_PARAM_STOCHASTIC.R")** This is the same as above, but the matrices are now stochastic and include the SE of the parameters. It uses truncated normal distributions and delivers SE on lambda after 1000 replicate simulated trajectories. *It is the script for the new Table S4.*

3) **source(“2_MR_2021_SCENARIOS.R”)**  It builds :

- The object “scenarios”, a matrix with 3 lines, corresponding to the frequency of drought at 0.1, 0.2 and 0.3 for 100 years. It stores the total n of animals each year according to the time-series considered. It does not contain replicates
- The object “N_overall” has the corresponding number of birds for each age and year. It begins with the age structure observed (tot=249):

N_overall[1:8,]<-c(30.45,30.45,6.96,6.96,6.96,6.96,42.63,42.63)

N_overall[9:16,]<-c(13.125,13.125,3,3,3,3,18.375,18.375)

- The object “ts_occur” is a series of 3 time series of 101 years built according to the frequency of sequia

4) **source("2tris_MR_2021_SEQUIA.R")** It builds an ARRAY ("all") with sets of 3x100x100 time series of droughts. This is for the envelop, 100 replicates (w). It also preduces “n.sequias” a matrix with the total number of droughts for each trajectory*. it delivers the array “all” and “n.sequias” for envelops*

5) **source("5_MR_2021_SEQUIA_NO_BAD_CONDITIONS.R")** it calculate trajectories of 100 populations (x3) with frequency 0.20. The first block considers only animals born in good conditions, the second block includes ALSO those born during drought years. **It builds a new array “scenarios” with the two natal conditions and one “scenarios_nb” without the carrying over effect. Only good conditions***. It is the script for FIG.4*
