# Supplementary material for: Hardship at birth alters the impact of climate change on a long-lived predator
Source: Nat Commun. 2022 Sep 27;13:5517. doi: 10.1038/s41467-022-33011-7 (PMC9515099; doi:10.1038/s41467-022-33011-7)
Supplement: Supplementary file 4 — Reporting Summary [file 41467_2022_33011_MOESM4_ESM.pdf]

nature research

Corresponding author(s): Fabrizio Sergio

Last updated by author(s): Aug 16, 2022

Reporting Summary

Nature Research wishes to improve the reproducibility of the work that we publish. This form provides structure for consistency and transparency in reporting. For further information on Nature Research policies, see our Editorial Policies and the Editorial Policy Checklist.

Statistics

For all statistical analyses, confirm that the following items are present in the figure legend, table legend, main text, or Methods section.

n/a	Confirmed	
☐	☒	The exact sample size (*n*) for each experimental group/condition, given as a discrete number and unit of measurement
☐	☒	A statement on whether measurements were taken from distinct samples or whether the same sample was measured repeatedly
☐	☒	The statistical test(s) used AND whether they are one- or two-sided<br>*Only common tests should be described solely by name; describe more complex techniques in the Methods section.*
☐	☒	A description of all covariates tested
☐	☒	A description of any assumptions or corrections, such as tests of normality and adjustment for multiple comparisons
☐	☒	A full description of the statistical parameters including central tendency (e.g. means) or other basic estimates (e.g. regression coefficient) AND variation (e.g. standard deviation) or associated estimates of uncertainty (e.g. confidence intervals)
☐	☒	For null hypothesis testing, the test statistic (e.g. F, t, r) with confidence intervals, effect sizes, degrees of freedom and P value noted<br>*Give P values as exact values whenever suitable.*
☒	☐	For Bayesian analysis, information on the choice of priors and Markov chain Monte Carlo settings
☒	☐	For hierarchical and complex designs, identification of the appropriate level for tests and full reporting of outcomes
☒	☐	Estimates of effect sizes (e.g. Cohen's *d*, Pearson's *r*), indicating how they were calculated

Our web collection on statistics for biologists contains articles on many of the points above.

Software and code

Policy information about availability of computer code

Data collection	No software was used for data collection
Data analysis	R.3.6.2; MARK 9.0

For manuscripts utilizing custom algorithms or software that are central to the research but not yet described in published literature, software must be made available to editors and reviewers. We strongly encourage code deposition in a community repository (e.g. GitHub). See the Nature Research guidelines for submitting code & software for further information.

Data

Policy information about availability of data

All manuscripts must include a data availability statement. This statement should provide the following information, where applicable:

- Accession codes, unique identifiers, or web links for publicly available datasets
- A list of figures that have associated raw data
- A description of any restrictions on data availability

The data generated in this study are available from Digital.CSIC at https://doi.org/10.20350/digitalCSIC/14705

Field-specific reporting

Ecological, evolutionary & environmental sciences study design

All studies must disclose on these points even when the disclosure is negative.

Study description	Statistical analysis of breeding success and survival data from a population of Red kites subject to long-term study
Research sample	Red kite population of Doñana National Park. This is a unique population where all ages of a very long-lived bird can be sampled, allowing unique insight into how life history traits can vary with conditions experienced as adults or at birth
Sampling strategy	The sample includes 701 individuals ringed as nestlings through several decades and then observed later on as adults, which allowed to adequately sample all age classes in the population
Data collection	The birds were ringed as nestlings and later observed as adults
Timing and spatial scale	Data were collected between 1986-2019. The whole study was conducted in Doñana National Park (southwestern Spain), i.e. over an approximate area of 1000 km2
Data exclusions	No data were excluded from analysis
Reproducibility	All attempts at reproducibility were successful
Randomization	The paper is not based on an experiment
Blinding	The paper is not based on an experiment

Did the study involve field work? ☒ Yes ☐ No

Field work, collection and transport

Field conditions	Fieldwork was implemented in Doñana National Park (south-western Spain), mainly during the period February-June and usually under calm days with mild temperatures (10-20 degrees C) and no rain
Location	Doñana National Park (36°56′51″N 6°21′31″O). Elevation = 1-33 m a.s.l
Access & import/export	The study was conducted in accordance with the national and European laws concerning the use of animals for scientific purposes (protocols EBD-11/09 and EBD-11/25 approved by the Animal Care and Bio-Ethics Sub-Committee of the Consejo Superior de Investigaciones Científicas - CSIC).
Disturbance	Upon marking, each nestling was quickly measured, ringed and returned to its nest. Each nestling was processed by experienced personnel in less than 5 minutes. Nest checks were always implemented quickly, so as to leave the area in less than 10 minutes in order to minimize disruption of breeding activities. All breeding individuals resumed their activities and there was no indication of an impact of human disturbance

Reporting for specific materials, systems and methods

We require information from authors about some types of materials, experimental systems and methods used in many studies. Here, indicate whether each material, system or method listed is relevant to your study. If you are not sure if a list item applies to your research, read the appropriate section before selecting a response.

Materials & experimental systems

n/a	Involved in the study
☒	☐ Antibodies
☒	☐ Eukaryotic cell lines
☒	☐ Palaeontology and archaeology
☐	☒ Animals and other organisms
☒	☐ Human research participants
☒	☐ Clinical data
☒	☐ Dual use research of concern

Methods

n/a	Involved in the study
☒	☐ ChIP-seq
☒	☐ Flow cytometry
☒	☐ MRI-based neuroimaging

Animals and other organisms

Policy information about studies involving animals; ARRIVE guidelines recommended for reporting animal research

Laboratory animals	n/a
Wild animals	We ringed 701 Red kites (Milvus milvus) nestlings and later observed tham as adults. Neslings were ringed in situ and immediately

	returned to their nest
Field-collected samples	The study did not involve laboratory samples collected in the field.
Ethics oversight	The study was conducted in strict accordance with the national and European laws concerning the use of animals for scientific purposes (protocols EBD-11/09 and EBD-11/25 approved by the Animal Care and Bio-Ethics Sub-Committee of the CSIC)

Note that full information on the approval of the study protocol must also be provided in the manuscript.
